# *De Novo* Genome Assembly of the Myanmar Puddle Frog, *Phrynoglossus myanhessei* (Anura: Dicroglossidae)

**DOI:** 10.7150/jgen.125510

**Published:** 2026-01-01

**Authors:** Katharina Geiß, Gunther Köhler, Axel Janke

**Affiliations:** 1 Senckenberg Forschungsinstitut und Naturmuseum, 60325 Frankfurt a.M., Germany.; 2 Senckenberg Biodiversity and Climate Research Centre (BiK-F), 60325 Frankfurt a.M., Germany.; 3 LOEWE-Centre for Translational Biodiversity Genomics (TBG), Senckenberg Nature Research Society, 60325 Frankfurt a.M., Germany.

**Keywords:** whole genome assembly, *Phrynoglossus myanhessei*, Dicroglossidae, Myanmar, holotype sequencing

## Abstract

The Myanmar puddle frog, *Phrynoglossus myanhessei*, is a recently described, small dicroglossid frog distributed across central and southern Myanmar, typically inhabiting areas adjacent to small stagnant water bodies. With that new species description, rudimentary genome data from 30-fold Illumina sequencing were published as a novel approach in taxonomy to routinely publish genome data for new holotypes. While the data allowed to assemble the entire mitochondrial genome, it was not possible to extract basic population genetic data. Therefore, we present a* de novo* PacBio CLR genome assembly of *P. myanhessei*, to aid population genomic, evolutionary and taxonomic studies. The assembled genome has a size of 2.28 Gbp, with a scaffold N50 of 44 kbp and largest scaffold being 270 kbp long. BUSCO analysis indicates a completeness score of 49%, with 26.9% complete and 22.3% fragmented BUSCOs. Approximately 43% of the genome consists of repetitive elements and about 22,500 genes could be predicted. While not an optimal assembly, the new *P. myanhessei* genome is a valuable resource for follow-up studies and for closing the gap in amphibian genome representation.

## Introduction

In addition to classical taxonomy, biodiversity is increasingly studied and described by Next Generation Sequencing (NGS) data analyses. However, genomic representation of species remains uneven even among vertebrates with most genome sequences derived from mammals (~4460 genomes) and birds (~2300 genomes) (National Center for Biotechnology Information; last accessed 21^st^ July 2025; 1-3). Amphibians are a rich tetrapod class with about 8900 species 4, representing a vertebrate class that includes the most threatened species, with nearly half of them being IUCN listed 5,6. As an ancient tetrapod lineage, they are globally distributed (except in the Arctic and Antarctica) and exhibit a unique diversity of traits, lifestyles, behaviours and reproductive strategies 7-12. Because of their rapid growth rate and high abundance, they serve as key components of food webs 13,14. These features make them attractive subjects for various scientific fields, including developmental biology, medical research, ecology and evolution 2,3. Despite their role of model organisms, they are still underrepresented in genomic studies utilizing NGS approaches 2,3.

To date, many studies on phylogenetic and taxonomic relationships of amphibians rely on a limited number of mitochondrial and nuclear markers 15-18. Recent genome sequencing initiatives, like the Earth Bio Genome project 19, produce and analyse large-scale genomic datasets. These allow for the investigation of genetic structures at increasingly broader geographical scales and higher resolution, even in non-model organisms such as amphibians 20,21, but have not reached momentum in this field. In addition, the assembly of amphibian genomes can often be challenging due to their large size and high repeat content 3,12,22, which are part of the reason for their underrepresentation among published genomes 1,2,23. Further limiting factors are the high costs and computational resources needed for analysing and assembling such genomes 24 as well as access to high-quality tissue for genome sequencing.

Compared to 4,400 mammalian genomes available on NCBI (National Center for Biotechnology Information; last accessed 3^rd^ July 2025) covering two-thirds of all known mammal species, over the past decade, only about 180 anuran genomes have been published. The majority of these are represented by the families Hylidae (16 genomes), Bufonidae (12 genomes) and Ranidae (10 genomes). This stark discrepancy highlights the need for high-quality genome assemblies particularly from underrepresented amphibian lineages.

**Figure 1:**
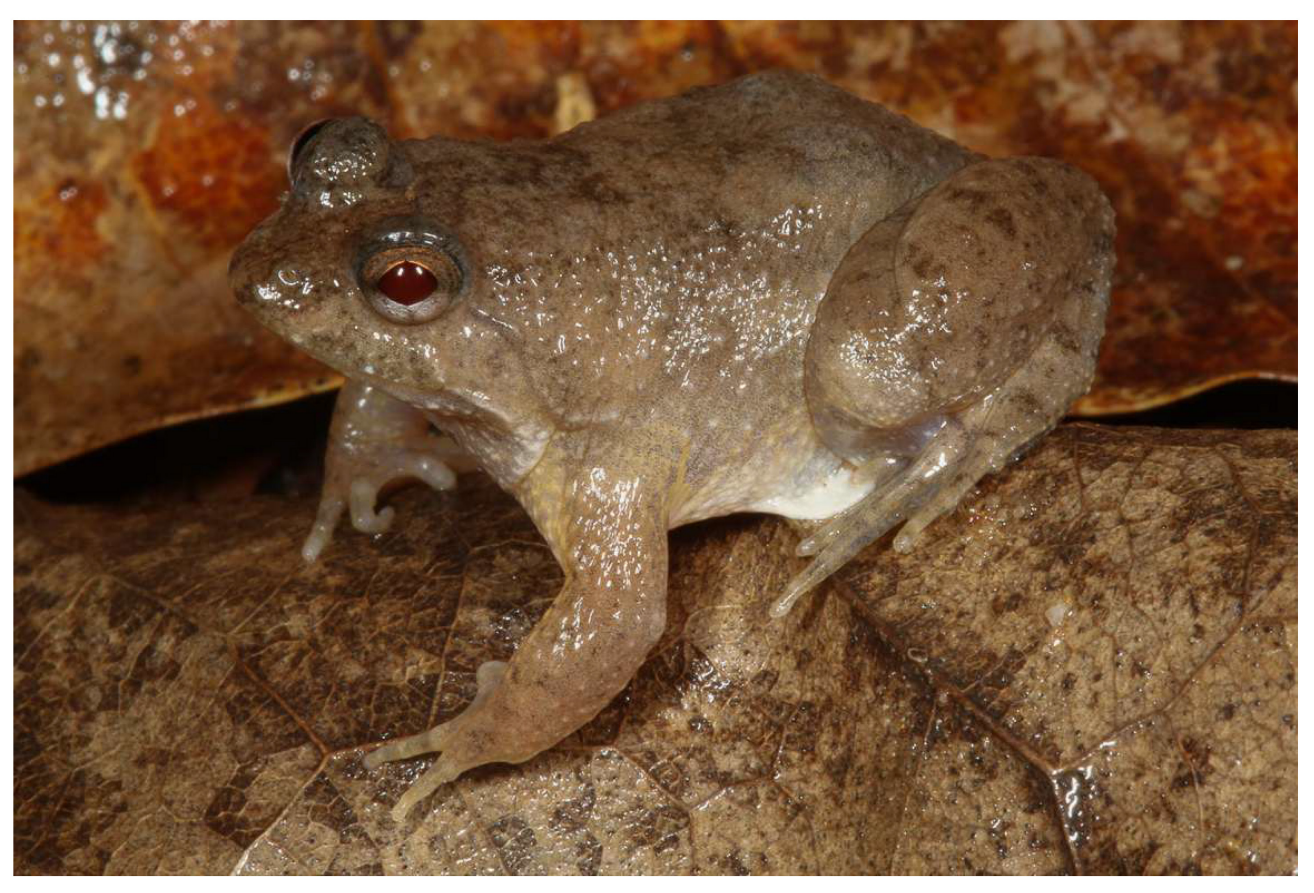
Male holotype of *Phrynoglossus myanhessei* (SMF 103841) in life. Specimen stored in the collection at Senckenberg Museum Frankfurt, Germany. Photo by GK published in Köhler *et al.* (2021).

The family Dicroglossidae, fork-tongued frogs, comprises a large group of frogs distributed from Sub-Saharan Africa through India to Southeast Asia, forming a significant component of local amphibian communities. It includes over 245 species 4, but genome assemblies are currently only available for four of them, three belonging to the subfamily Dicroglossinae (*Hoplobatrachus occipitalis*, *Nanorana parkeri* and *Quasipaa spinosa*) and one to the subfamily Occidozyginae. Within the latter, only one rough draft genome based on 30-fold Illumina sequences is available for *Phrynoglossus myanhessei* (Figure 1). This species has been recently described from Myanmar 25 belonging to the genus *Phrynoglossus* (Peters 1867). Members of this genus are characterized by a fleshy and swollen tongue, the absence of vomerine teeth, slightly swollen digit tips, a distinct tympanum, skin covered by extensive mucous (“slimy touch”), a grey throat and both an axillar and inguinal amplexus 25,26. They are semiaquatic animals that tend to sit at the edge of small and shallow temporary water bodies 4,25. *P. myanhessei* remains poorly understood in terms of its natural history and ecology. Its known distribution is currently restricted to the central and southern regions of Myanmar, with no records from the Malay Peninsula 4.

The available genomic data of *P. myanhessei* were published in 2021 as a genome resource (1.8 Gbp) with high fragmentation (N50 1.5 kbp) and low completeness (BUSCO: 8.5%). They were released alongside its taxonomic description to provide basic genomic information for a newly described holotype and to support future research 25. As proof of principle to promote type-specimen genomics 27, the mitochondrial genome and a few nuclear genes could be identified in the original publication. The 30-fold Illumina paired-end coverage was an economic way to document its entire genome as a routine dataset for new species descriptions of new holotypes. However, its deeper information could only be extracted by mapping to a reference genome.

To improve the genome quality of this holotype, we generated Pacific Biosciences (PacBio) long-reads and applied Illumina short-read error correction, resulting in an improved reference genome for this genus.

## Materials and Methods

### Taxon sampling

The sequenced male specimen of *Phrynoglossus myanhessei* (field number GK 6728; museum voucher SMF 103841) was collected by Gunther Köhler (GK) on 6 July 2017 at East Yangon University, Yangon Province, Myanmar (16.77737N, 96.24065E, WGS 1984). The specimen is stored at the herpetological collection at the Senckenberg Museum, Frankfurt (SMF), Germany.

### Genomic library preparation

The protocols for DNA extraction and preparation for Illumina short-read sequencing are described in detail in Köhler *et al.* (2021) [25]. The short reads were deposited by Köhler *et al.* (2021) [25] under the accession number *SRR13288470*.

For PacBio consensus long read (CLR) sequencing genomic DNA was extracted from 2.5 mg tongue tissue following the standard phenol chloroform protocol 28. The obtained DNA was resuspended in TE buffer (10 mM Tris Cl, 0.1 mM EDTA) and stored at -20 °C. Quality of the extracted DNA was assessed using TapeStation 2200 from Agilent Technologies 29.

### Genome assembly and scaffolding

Based on the raw sequencing data, a k-mer profile was generated using Jellyfish 2.3.0 30 and visualized with GenomeScope 2.0 31,32. The quality of the raw reads was assessed using FastQC 0.11.9 33. Adapter sequences and low-quality bases were removed using Trimmomatic 0.39 34.

The obtained long reads of SMF 103841 were assembled using Flye 2.9.2 with the pacbio-raw flag and an estimated genome size of 2.5 g 35. The assembly was polished with long reads using Racon 1.5 36 and deduplicated short reads using Pilon 1.24 37. To clean the short reads before polishing, they were mapped against the assembly using BWA 0.7.17 38 and Samtools 1.17 39. Additionally, duplicates were marked using Picard 3.0 40. To increase continuity, long-read scaffolding was performed with LongStitch 1.0.5 41. To improve the correctness of the scaffolded assembly, gap closing was conducted with TGS-GapCloser 1.2.1 42.

### Assembly quality assessment

Contiguity and basic statistical data of the obtained assembly were assessed using QUAST 5.2.0 43. To check the assembly for contamination by other organisms, the contigs were aligned with the NCBI database using blastn algorithm 44. The results were visualized with Blobtools 1.1.1 using the “bestsum” algorithm 45. Contigs assigned to lineages other than vertebrates were checked for vertebrate BUSCOs using BUSCO 5.4.3 with the tetrapoda orthologous gene set (tetrapoda_odb10) 46. Contigs were maintained for the assembly when vertebrate BUSCOs were detected. To improve the correctness of the obtained assembly, contigs smaller than 500bp were removed. In addition, contigs were aligned with the NCBI database using blastn algorithm to remove contigs assigned to the mitochondrial genome 44. Furthermore, BUSCO 5.4.3 was used to evaluate the completeness of the assembled genome using the vertebrate orthologous gene set (vertebrata_odb10) 46.

### Genome annotation

***Repeat annotation:*** Repeat annotation was performed using RepeatModeler 2.0.4 and RepeatMasker 4.1.5 47,48. First, a species-specific repeat library was generated with RepeatModeler 2.0.4 (using NCBI rmblast 2.14.0+ engine) including RECON 1.08 49, RepeatScout 1.0.6 50, LTRharvest 51 and LTR_retriever 52. This library was then combined with lineage-specific repeats from Dfam 53 using *famdb.py* to create a custom library. Repeat masking was performed with RepeatMasker using the combined library, applying both hard and soft masking. Finally, the repeat landscape was plotted based on Kimura 2-parameter divergence using RepeatMasker utilities.

***Gene annotation:*** Protein-coding gene models were predicted using GeMoMa 1.9 54, which transfers gene annotations from multiple reference species to the target assembly via homology-based projection and intron position conservation. The genome was annotated with the GeMoMa pipeline, using annotations and genomes from seven amphibian references (*Bufo bufo* GCF_905171765.1, *Bufo gargarizans* GCF_014858855.1, *Hyla sarda* GCF_029499605.1, *Nanorana parkeri* GCF_000935625.1, *Rana temporaria* GCF_905171775.1, *Xenopus laevis* GCF_017654675.1 and *Xenopus tropicalis* GCF_000004195.4). GeMoMa was run with realignment enabled (GeMoMa.Score=ReAlign) to output predicted coding sequences and proteins. Statistics of the predicted proteins were summarized using AGAT 55. The final protein set was assessed for completeness using BUSCO 5.4.3 in “protein” mode with the vertebrate ortholog set (vertebrata_odb10) 46.

### Variant calling and demographic inference

Short reads were mapped to the final assembly using BWA-MEM 0.7.17 38 and duplicate reads were removed using “MarkDuplicates” from Picard 3.0.0-1 40. Mapping quality was assessed with Qualimap 2.2.1 56.

Samtools 1.19 was used to calculate site depth statistics 57. Variant calling was done using bcftools 1.19 “mpileup” and “call -m” 39. Variants were filtered based on read depth (DP) using bcftools filter, retaining only those with DP between 30 and 75 to exclude low-confidence and highly covered sites. Genome-wide variant statistics were obtained using bcftools “stats”. Genome-wide heterozygosity (H_E_) was calculated from variant statistics as the proportion of heterozygous genotypes relative to total genotypes. The genome-wide genotype error rate was estimated as the proportion of non-reference homozygous calls relative to the total number of genotype sites obtained from the variant statistics to assess sequencing accuracy. We estimated effective population size (N_e_) based on H_E_ and mutation rate (μ) using the formula:

N_e_=H_E_ / (4×μ)

We based our mutation rate on synonymous substitution rates reported by Session *et al.* (2016) [58] for *Xenopus laevis*, who estimated an absolute substitution rate of approximately 3.0 × 10⁻⁹ substitutions per site per year, excluding CpG sites. Assuming a generation time of 2 years for *X. laevis*, this translates to a per-generation mutation rate of approximately 6.0 × 10⁻⁹ (0.6 × 10⁻⁸) substitutions per site per generation. However, because direct estimates for our study species are unavailable, and to account for variation in mutation rates among amphibians and vertebrates more broadly, we also considered a plausible range of 0.5-1.0 × 10⁻⁸ mutations per site per generation 59,60.

### Data availability

The genome assembly generated during this project is accessible on GenBank (Bioproject PRJNA687006; Accession No. JBRATO000000000). Supplemental material available at Journal of Genomics online.

## Results

### Genome sequencing and assembly

High quality genomic DNA with an average length of >10kbp could be extracted from tongue tissue (see Supplementary Figure 1). Since the sample has been stored in Ethanol for ~5 years, it was not suitable for RNA isolation and generation of a transcriptome.

PacBio CLR sequencing produced 119 Gb of long read data with a mean read length of 8,047 bp, a total of 7,910,035 reads and a total length of 63,653,327,111 bp. Illumina short-read sequences yielded two identical files of 211 Gb short-read data, each containing 308,075,534 reads with a total length of 46,211,330 bp (Table 1).

The *de novo* assembly of *Phrynoglossus myanhessei* from PacBio and Illumina data resulted in a genome of 2.28 Gbp consisting of 70,197 scaffolds, with a scaffold of N50 of 44 Kbp and L50 of 16,441 bp (Table 2). Estimated long-read and short-read coverage of the assembly were 14.6× and 13.3×, respectively.

Blobtools classified 73% of scaffolds as Chordata, ~1% as arthropoda and 7% unknown (see Supplementary Figure 2). BUSCO analysis of the cleaned assembly recovered a total of 49.2% BUSCOs, including 26.9% complete (Table 2).

### Genome annotation

**Figure 2:**
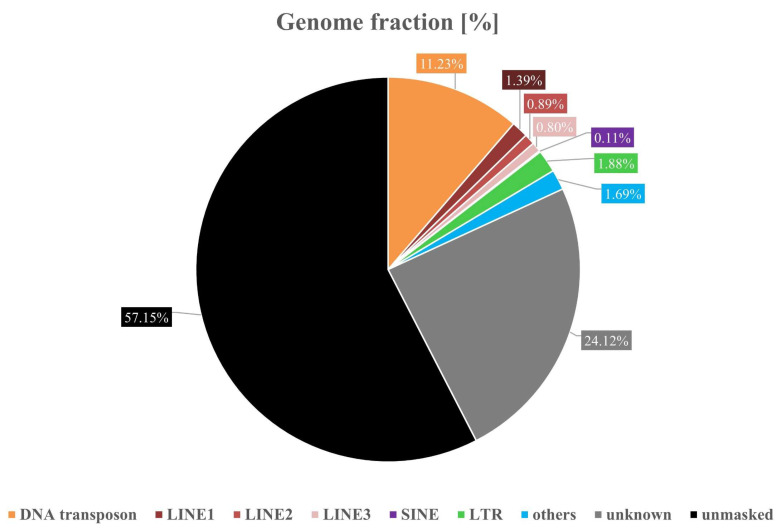
Genome fraction of the *Phrynoglossus myanhessei* (SMF 103841) de novo assembly showing the proportion of repetitive elements (with the black area representing the non-repetitive portion of the genome).

Repeat masking identified 42.9% of the de novo genome as repetitive elements (RE) (Figure 2). Of these, 5.23% were classified as retroelements, including 0.08% SINEs, 3.08% LINEs (1.39% LINE1, 0.89% LINE2 and 0.8% LINE3), and 1.88% LTR elements. DNA transposons accounted for 11.23%, rolling-circle elements for 0.05%, small RNAs for 0.03%, satellites for 0.13% and simple repeats for 1.53%. Notably, 24.12% of the repetitive sequences could not be assigned to known classes.

Genome annotation, combining de novo and homology-based repeat identification, resulted in 22,508 genes, 29,402 mRNAs, and 175,281 coding sequences (CDSs). The annotated genes had an average length of 9,168 bp. Each CDS is composed of an average of 5.5 exons and 4.5 introns with mean exon and intron lengths of 210 bp and 1777 bp. BUSCO analysis of the predicted proteins identified 31.0% complete proteins, of which 8.2% were duplicated. Additionally, 16.4% of the predicted proteins were fragmented and 52.6% were missing (total n=3352).

### Variant calling and demographic inference

Variant Calling recovered about 74.6 million sites, of which 74.3 million were monomorphic and about 268,000 were biallelic variants. Genome-wide heterozygosity (Ho) was estimated at 0.358%. Genotype calls were highly accurate, with a low estimated genotype error rate of about 0.0019%.

Assuming mutation rates ranging from 0.5 × 10⁻⁸ to 1.0 × 10⁻⁸ per site per generation, effective population size (Ne) was estimated to range from approximately 90,000 to 180,000 individuals, indicating substantial genetic diversity in the studied population.

## Discussion

For more than 250 years, species descriptions in taxonomy have relied on physical specimens—specifically the holotype—which is collected, examined, described in published literature, and permanently deposited in natural history museum collections 27. Taxonomic comparisons, subspecies delimitations and assessments of closely related taxa require access to these name-bearing specimens, the holotype 27,61,62. This often necessitates either complicated loans of the specimen or travel to holding institution. However, over time, type specimens inevitably deteriorate: colors fade, anatomical features shrink, and fur or feathers may be lost 27.

Genomic data provide a permanent and globally accessible complement to traditional type material. Generating 20-30x short-read coverage of a genome providing comprehensive data that is inexpensive in comparison to the logistics of field collection and long-term specimen curation. However, genomic data requires decoding through mapping to a reference genome of a closely related species, which is often unavailable for non-model organisms, such as in our case *Phrynoglossus myanhessei*.

For more in-depth taxonomic, population genomic, or evolutionary studies, a draft reference genome becomes essential. Such a genome enables the retrieval of protein-coding genes, estimation of heterozygosity, and reconstruction of demographic history 27. Additionally, the reference genome can serve as a scaffold to map short reads from closely related individuals, facilitating accurate species delimitation—one of the fundamental goals of taxonomy.

Here, we present an improved genome assembly of *Phrynoglossus myanhessei*. However, the assembly remains non-contiguous, as measured by scaffold N50 and L50 metrics. High heterozygosity complicates genome assembly, even with long-read platforms such as PacBio, due to the increased presence of alternative haplotypes. This often leads to fragmented assemblies with reduced contiguity, reflected in lower N50 values 63,64.

Additional factors might likely contribute to the limited contiguity and completeness of the assembly, including only moderate DNA integrity (despite >15 kb fragments), the reliance on PacBio CLR and short-read Illumina data, and the absence of RNA-seq or linked-read data. While high quality DNA with a length of >15kpb could be extracted from the material suitable for PacBio CLR sequencing, the material did not allow for additional RNA sequencing due to its nearly five years of ethanol preservation and frozen storage. The sequencing strategy of using PacBio CLR in combination with Illumina short-reads and the resulting read data are limiting read length and quality compared to high-quality assemblies that mainly rely on including transcriptome or linked-read technologies. The short-read Illumina data although having high read quality are insufficient in resolving repetitive regions and structural complexity and lead to fragmentation and gaps in the assembly 65.

The relatively low BUSCO values (~50%) likely underestimates the true completeness of this novel genome assembly. BUSCO analysis was conducted using a general vertebrate database rather than one tailored to amphibians or anurans 66,67. This bias can lead to underestimating predicted gene or protein completeness. However, when compared with other amphibian genome assemblies generated using similar sequencing and analysis approaches, our results are consistent, typically showing ~20% lower completeness than highly scaffolded or chromosome-level assemblies 68-71.

This reference bias also extends to repeat annotations: when homology-based repeat libraries are incomplete, true genes may be misclassified as repetitive elements, artificially increasing the proportion of “unknown” repeats 3,72. In our genome assembly, the total repeat content is slightly lower than that reported for other dicroglossid anurans (47-64%) yet remains well within the known range documented for anurans (23-82%) 12. In contrast, the initial draft assembly of *P. myanhessei* (GCA_022657655.1) showed a higher repeat content (47.65%), likely a consequence of the greater fragmentation of the assembly, which can lead to an overestimation of repetitive samples.

Moreover, our study reveals that *P. myanhessei* exhibits relatively high heterozygosity and effective population size compared to other amphibians. Threatened species in particular often exhibit reduced heterozygosity, due to often small and fragmented populations and limited connectivity among them 73,74. Likewise, formerly widespread amphibians such as the boreal toad (*Anaxyrus boreas*) now display markedly lower heterozygosity and nucleotide diversity, likely reflecting historical bottleneck effects and recent population declines 75-77. In contrast, heterozygosity in *P. myanhessei* (~0.35%) is comparable to that of the African clawed frog (*Xenopus tropicalis*) (~0.3%), a widely distributed species without signs of demographic decline 78.

The relatively high heterozygosity in *P. myanhessei* suggests a large and stable population. Field observations support this interpretation: the species is common across its distribution range, readily occupying both natural and anthropogenic habitats, and breeds opportunistically, with males calling from any suitable puddle (GK pers. comm.). Such ecological traits facilitate gene flow within a population and thus help maintain genetic diversity by mitigating the impacts of genetic drift and inbreeding 79-81.

The genome annotation performed with GeMoMa predicted approximately 22,000 protein-coding genes, which lies within the expected range for vertebrate genomes 82. Despite the fragmented nature of the assembly, this relatively high gene count underscores the value of homology-based gene prediction tools, which can recover conserved gene models by aligning the fragmented scaffolds to a reference genome of a related species 83. However, the high fragmentation still limits annotation accuracy and completeness, contributing to the relatively low BUSCO completeness score (~30%). This likely reflects methodological constraints, such as fragmentation of gene models, partial or misassembled exons, and limitations of the available reference databases, rather than true biological absence. Consequently, many functional genes are likely present in the genome but were not detected or fully annotated by BUSCO.

Despite these limitations, the assembly remains suitable as a reference for mapping-based analyses, including population genomics and variant calling. Assemblies of comparable quality have been successfully used in other anuran population genomic studies, such as the *Phyllomedusa burmeisteri* species group 71. The recovered protein-coding genes therefore remain valuable for exploring controversial phylogenetic relationships within the subfamily Occidozyginae.

## Supplementary Material

Supplementary figures.

## Figures and Tables

**Table 1:** Summary statistics of the raw 1 and the filtered and trimmed 2 Illumina short read data of *Phrynoglossus myanhessei* SMF 103841.

**(1) Statistics of raw short reads**		
No. of short reads	308,075,534	
Average read length [bp]	150	
Total length [bp]	46,211,330,100	
Duplicates [%]	25.3	
GC [%]	42	
**(2) Statistics of trimmed short reads**		
	forward	reversed
No. of short reads	301,535,351	301,535,351
Average read length [bp]	150	150
Total length [bp]	45,227,130,304	45,226,425,599
Duplicates [%]	24.9	24.3
GC [%]	42	42

**Table 2:** Summary statistics of the *Phrynoglossus myanhessei* SMF 103841 scaffold-level reference genome. Details on assembly statistics 1 and BUSCO analysis 2 are shown.

(1) Statistics of long reads			
		contigs	scaffolds
Total no.		77,560	70,197
Total length [bp]		2,407,983,688	2,279,771,963
Largest contig [bp]		289,698	271,019
N50		42,693	44,633
L50		18,255	16,441
GC [%]		42.72	42.7
Ns per 100 kbp		0	185.5
No. of total Ns		0	4,229,927
Mean long read coverage [x]		14.6	
Mapped long reads [%]		92.1	
Mean short read coverage [x]		13.3	
Mapped short reads [%]		85.6	
**(2) BUSCO completeness (n = 3354)**			
S: 26.6%	D: 0.3%	F: 22.3%	M: 50.8%
			

## Abbreviations

BUSCO: Benchmarking Universal Single Copy Orthologs
CDS: coding sequences
CLR: consensus long read (sequencing)
GeMoMa: Gene Model Mapper
GK: Gunther Köhler
H_E_: heterozygosity
LINE: long interspersed nuclear elements
LTR: long terminal repeats
N_e_: effective population size
NGS: Next Generation Sequencing
RE: repetitive elements
SINE: short interspersed nuclear elements
SMF: Senckenberg Museum Frankfurt
