# Supplementary material for: De Novo Genome Assembly of the Myanmar Puddle Frog, Phrynoglossus myanhessei (Anura: Dicroglossidae)
Source: J Genomics. 2026 Jan 1;14:1–9. doi: 10.7150/jgen.125510 (PMC12766079; doi:10.7150/jgen.125510)
Supplement: Supplementary file 1 — Supplementary figures. [file jgenv14p0001s1.pdf]

Supplementary Figures

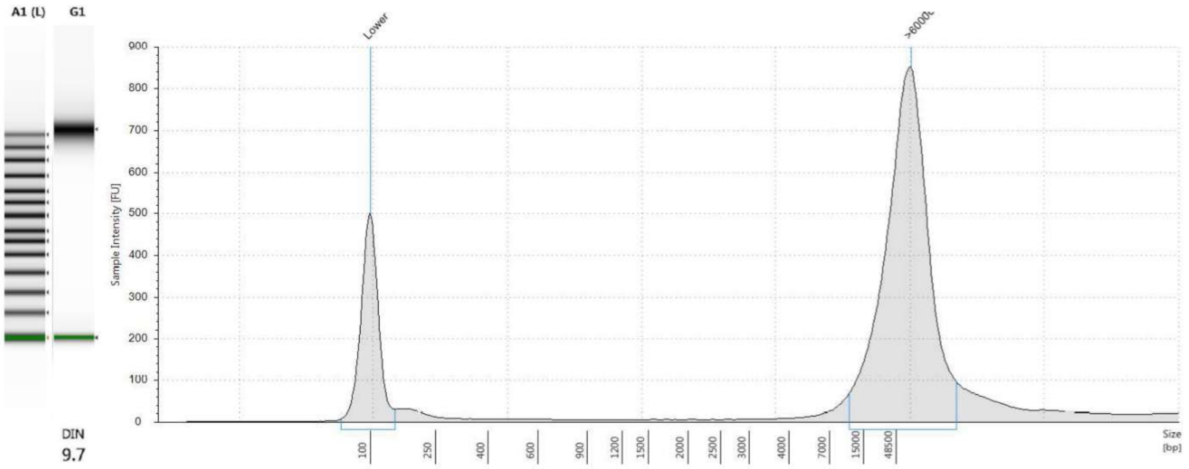

Supplementary Figure 1 TapeStation results of sample *Phrynoglossus myanhessei* (SMF 103841) showing the fragment size distribution as a gel image (left) and an intensity plot (right). The plot illustrates fragment sizes (bp) versus fluorescence intensity (FU). A1 = Ladder. The TapeStation analysis reported a DNA Integrity Number (DIN) of 9.7 and a total concentration of 47.5 ng/µl. The major peak was observed above 10,823 bp, representing 87.05% of the integrated area, with a calibrated concentration of 35.6 ng/µl, indicating high molecular weight DNA.

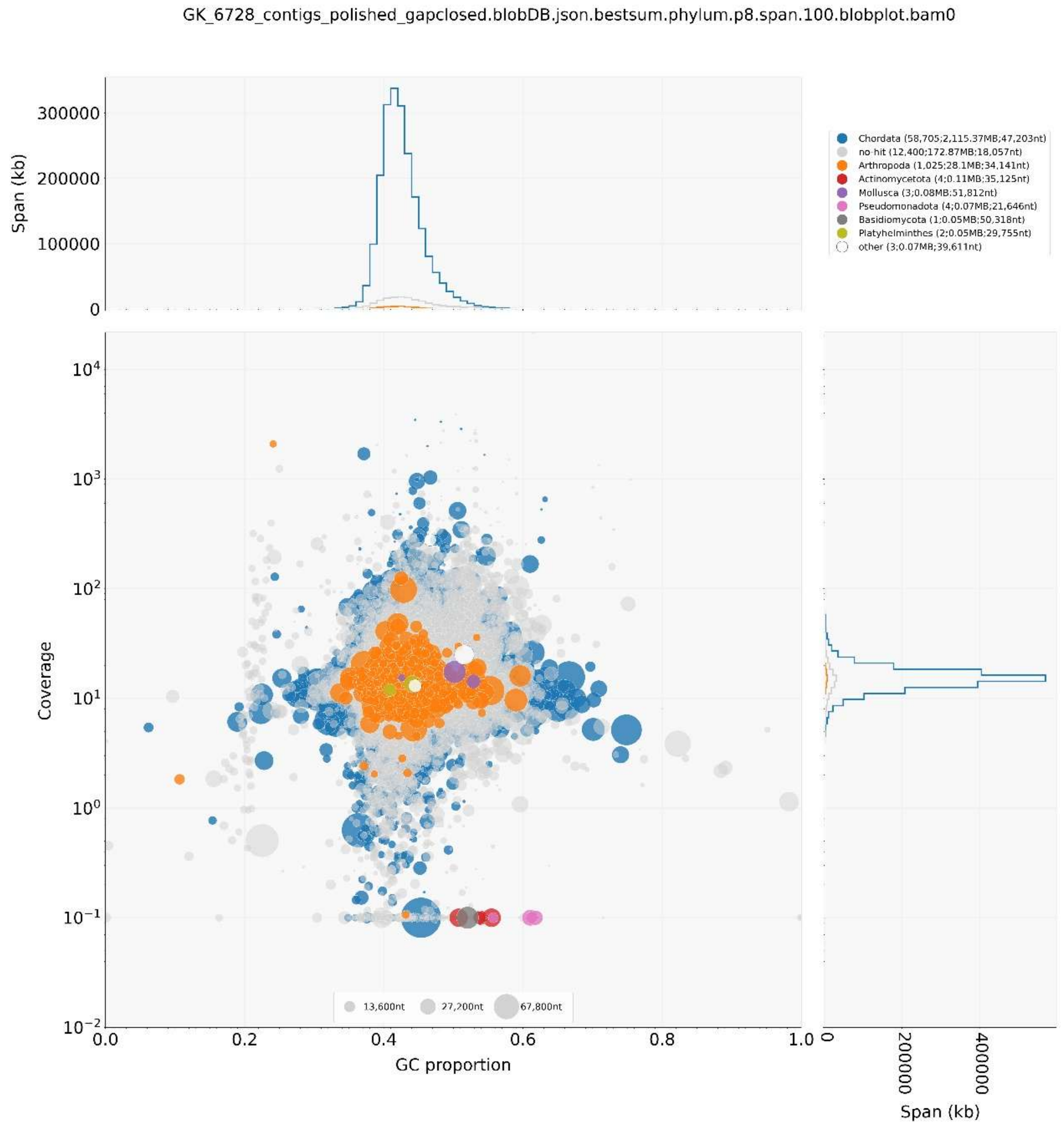

Supplementary Figure 2 Blobplot of *Phrynoglossus myanhessei* (SMF 103841) genome assembly showing amount of contamination. The plot illustrates GC proportion versus coverage for each scaffold. Scaffolds that were kept for analysis are illustrated in blue (Chordata; 73.40%) and grey (no-hit = unassigned; 7.10%). Contamination that has been removed from assembly has been assigned to Arthropoda (0.98%) in orange, Actinomyceota (< 0.01%) in red, Mollusca (< 0.01%) in purple, Pseudomonadota (< 0.01%) in pink, Basidiomycota (< 0.01%) in dark grey, Plathelminthes (< 0.01%) in green and others (< 0.01%) in white.
